# Comparative Study of Transcriptome in the Hearts Isolated from Mice, Rats, and Humans

**DOI:** 10.3390/biom12060859

**Published:** 2022-06-20

**Authors:** Daigo Okada, Yosuke Okamoto, Toshiro Io, Miho Oka, Daiki Kobayashi, Suzuka Ito, Ryo Yamada, Kuniaki Ishii, Kyoichi Ono

**Affiliations:** 1 Center for Genomic Medicine, Graduate School of Medicine, Kyoto University, Shogoinkawahara-cho, Kyoto 606-8507, Japan; dokada@genome.med.kyoto-u.ac.jp (D.O.); yamada.ryo.5u@kyoto-u.jp (R.Y.); 2 Department of Cell Physiology, Akita Graduate School of Medicine, Hondo, Akita 010-8543, Japan; dkobayashi@med.akita-u.ac.jp (D.K.); s4020512@s.akita-u.ac.jp (S.I.); onok@med.akita-u.ac.jp (K.O.); 3 Research Department, Ono Pharmaceutical Co., Ltd., Kyutaromachi, Osaka 618-8585, Japan; io@ono.co.jp (T.I.); m.oka@ono.co.jp (M.O.); 4 Department of Pharmacology, Faculty of medicine, Yamagata University, Iida-Nishi, Yamagata 990-9585, Japan; kuishii@med.id.yamagata-u.ac.jp

**Keywords:** transcriptome, heart, SHOX2

## Abstract

The heart is a significant organ in mammalian life, and the heartbeat mechanism has been an essential focus of science. However, few studies have focused on species differences. Accordingly, challenges remain in studying genes that have universal functions across species and genes that determine species differences. Here, we analyzed transcriptome data in mouse, rat, and human atria, ventricles, and sinoatrial nodes (SA) obtained from different platforms and compared them by calculating specificity measure (SPM) values in consideration of species differences. Among the three heart regions, the species differences in SA were the greatest, and we searched for genes that determined the essential characteristics of SA, which was *SHOX2* in our criteria. The SPM value of *SHOX2* was prominently high across species. Similarly, by calculating SPM values, we identified 3 atrial-specific, 11 ventricular-specific, and 17 SA-specific markers. Ontology analysis identified 70 cardiac region- and species-specific ontologies. These results suggest that reanalyzing existing data by calculating SPM values may identify novel tissue-specific genes and species-dependent gene expression. This study identified the importance of *SHOX2* as an SA-specific transcription factor, a novel cardiac regional marker, and species-dependent ontologies.

## 1. Introduction

The heart is the principal organ of mammalian lives, which functions first in an early developmental stage, and never stops for the rest of life. Thus, the molecular processes underlying heart function have been a significant focus of science for a long time. From the 1950s to the 1970s, actin-myosin sliding filament theory revealed the contraction mechanisms in response to intracellular Ca^2+^ ([Ca^2+^]_i_) elevation accompanied by the membrane depolarization via voltage-gated ion channels [1,2,3]. From the 1980s to the 1990s, molecular cloning strategies identified the gene sequences and primary structures of the ion channels [4,5,6,7]. The hyperpolarization-activated current (I_h_) in the sinoatrial node (SA) was recorded in 1976 [8], which become known as the I_f_ current [9] via the HCN4 channel [10]. It is thought that [Ca^2+^]_i_ initiates the SA beating [11] as well as the ventricular contraction. These cardiac activities are regulated by the autonomic nervous system. One is the sympathetic control with the adrenergic stimulation. Intracellular signaling under the β-adrenaline receptor reinforces the ion currents and [Ca^2+^]_i_ movements, increasing the contraction’s capability and accelerating the heart rate. The other is the parasympathetic control with the cholinergic signaling, which oppositely suppresses the sympathetic effects [12,13]. As such, muscle filament, ion channels, Ca^2+^ handling, and cell signaling networks are regarded as fundamental factors in cardiac physiology.

However, while the essential functions of the heart are believed to be well established, biophysical cardiac parameters at the molecular and cellular levels are not consistent throughout the species. For example, a Na^+^-Ca^2+^ exchanger (NCX) is a device that converts [Ca^2+^]_i_ elevation into membrane potential, which is essential for driving SA automaticity in rabbits and mice, as mentioned above. Meanwhile, the guinea-pig SA beats independently of changes in [Ca^2+^]_i_ [14] and is not suppressed by an NCX inhibitor [15]. The L-type Ca^2+^ current (LCC) responses to intracellular cGMP differ in guinea-pigs, rats, and humans. cGMP magnifies the β-adrenergic LCC enhancement in guinea-pigs [16] while it exhibits the opposite effect in rats [17]. Further study using human myocytes uncovered that cGMP itself increased LCC without other effectors. Rivet-Bastide et al. proposed that different actions of cGMP on LCC were mainly attributable to the difference in the phosphodiesterase isotype [17]. In the case of potassium current, the 4-aminopyridine sensitive transient outward current observed in mouse, rat, and human cardiomyocytes [18,19] is unrecognizable in guinea-pigs [20]. By contrast, an isoprenaline-stimulable Cl^-^ current of the heart is prominent in guinea-pigs rather than in other mammals [21,22]. A comparative study of I_h_ was recently demonstrated in rat, guinea pig, and rabbit pulmonary vein cardiomyocytes [23,24]. Surprisingly, I_h_ in rats is neither I_f_ nor I_KH_ [25], but ClC-2 current [26]. In this careful and refined consideration of the data, species differences in molecular function within the heart have generally been observed. Therefore, a comprehensive comparison of species differences in molecular information within the heart is a critical challenge for future cardiac research. Thus, to compare species differences in gene expressions in a high-throughput manner, we acquired unpublished transcriptome data. We acquired these data from academic institutions or a pharmaceutical company that had launched experiments and remained unpublished because they failed to achieve their original purposes.

In the current study, we present an analysis of the transcriptomes in three different animal species—mice, rats, and humans—separately for atria, ventricles, and SA. The bioinformatical evaluation across the species disclosed the essential genes of the heart and the crucial difference among the species.

## 2. Materials and Methods

### 2.1. RNA Extractions from Experimental Animals

In order to extract endogenous RNAs from different parts of cardiac tissues, 16 C57/BL6 male mice (5–10 weeks) and 12 Wistar male rats (8–11 weeks) were sacrificed. Three mice and two rats were required to prepare an adequate RNA sample for the sinoatrial node (SA). In the surgical operation, the animals were deeply anesthetized by 320–450 mg/kg pentobarbital sodium with 100 UI heparin. After checking suppression of the nociceptive reflex, the chest cavity was opened, and the heart and lungs were excised in a block and then perfused sequentially with ice-cold, Ca^2+^ free, and heparinized external solution until most of the blood was washed out from the heart and lungs. The composition of the external solution (mM) was: NaCl 136.9, KCl 5.4, NaH_2_PO_4_ 0.33, HEPES 5.0, MgCl 0.5 and glucose 5.5 (pH 7.4 with NaOH). The heart-lung block was pinned to the tissue bath. Soft tissue containing the vagus nerve and adipose tissue was trimmed off under a stereomicroscope, then cardiopulmonary tissues were isolated one by one. The left atrium (LA) adjacent to the pulmonary vein (PV), a mass of the left ventricle (LV), and the free-wall of the right ventricle (RV) were isolated. Each LV mass was dissected into three pieces as samples. Because the SAN isolation procedure takes approximately 20 min, SA was isolated separately. The SA region was delimited by the borders of the crista terminalis, the interatrial septum, the superior vena cava, and the right atrium (RA) (Supplementary Figure S1). Our SA-cutting technique was confirmed by quantitative PCR for mice and by the microarray data itself for rats (Supplementary Figure S2). Because the SAN is a tiny cardiac area, three mice and two rats were needed to extract the required amount of RNA for the microarray. All cardiopulmonary tissues were isolated within 30 min after the heart was removed from the body. During tissue isolation, the external solution was perfused at a rate of 10 mL/min on ice. In addition to PV, LV, RV, SA, and RA samples, pulmonary arteries were added to the samples for the rat microarray. All tissue samples were fresh-frozen in liquid nitrogen and stored at −80 °C for later RNA extraction. RNAs were extracted using QIAGEN RNeasy mini columns (QIAGEN, Venlo, The Netherland).

### 2.2. RNA Extractions from Heart Donations

Human heart samples were purchased from AnaBios Corporation (San Diego, CA, USA), which provides heart organs through the organ procurement organization of the USA in compliance with the Health Insurance Portability and Accountability Act. All personal information of donors is protected. In the current study, three normal hearts were separated into LA, RA, LV, RV, and SAN. These samples were frozen in RNAlater (Thermo Fisher Science, Waltham, MA, USA) at −20 °C. RNAs were extracted in our laboratory using QIAGEN RNeasy mini columns (QIAGEN).

### 2.3. Mouse Gene Expression Microarrays (Illumina)

Five hundred ng total RNA was labeled according to the manufacturer’s instructions using the Illumina TotalPrepTM RNA amplification kit (Illumina, San Diego, CA, USA), and 750 ng biotinylated RNA per sample hybridized overnight to Illumina Mouse Ref-8 v2 BeadChips. Following post-hybridization rinses, arrays were incubated with streptavidin-conjugated Cy3, and scanned at a resolution of 0.53 μm using an Illumina iScan scanner. Hybridization intensity data were extracted using Illumina BeadStudio GenomeStudio software, ver. 2011.1 (Illumina).

A total of 18 microarray data samples were used for the mouse data, consisting of 4, 2, 4, 4, 4 samples of LA, LV, RA, RV, and SA. Signal values were transformed by log2(intensity + 1) and standardized using the normalizeBetweenArrays function in the limma package to align the medians of all arrays [27]. The probe information was converted to gene information corresponding to unique Ensemble Gene IDs. This conversion was done via Entrez Gene ID using DAVID web tool [27]. When multiple probes corresponded to a single gene, the maximum signal intensity value was adopted.

### 2.4. Rat Gene Expression Microarrays (Agilent)

RNAs from rat samples were amplified as complementary RNA (cRNA) and labeled with Cy3 using the Low-Input QuickAmp Labeling kit (Agilent Technologies, Santa Clara, CA, USA). Cy3-labeled cRNA was fragmented and hybridized to SurePrint G3 Rat GE microarray 8 × 60K v2 (Agilent Technologies). SurePrint Array was scanned by the Agilent DNA Microarray Scanner (G2505C).

A total of 23 microarray data samples were used for the rat data, with 4, 4, 4, 4, 4, 4, 3, 4 samples of LA, LV, PA, PV, RV, and SA. All background-subtracted data were detrended and normalized by 90 percentile normalization. Probes containing missing values were removed, and probe information was converted to gene information corresponding to unique Ensemble Gene IDs. This conversion was done via the Ensemble Transcriptome ID. Here, when multiple probes corresponded to a single gene, the maximum signal intensity value was adopted. The gene expression signal values thus obtained were converted by log2(intensity + 1) and used as gene expression intensity in subsequent analyses.

### 2.5. RNA-Seq on the Donated Hearts

The high-throughput sequence was demonstrated with NovaSeq 6000 (Illumina). First, extracted RNAs were purified by poly(A) capture. Resultant mRNAs were then fragmented and reverse-transcribed into single-stranded complementary DNAs (cDNAs). Subsequently, cDNAs were double-stranded by a DNA polymerase. During the polymerase reactions, deoxy UTP (dUTP) was mixed in nucleotide materials. Both ends of double-stranded DNA (ds DNA) were ligated to a 13 bp adapter sequence. Next, the ds DNAs were subjected to PCR amplification for the multi-sized DNA library preparation. NovaSeq Control software v1.4.0 analyzed the sequencing runs and tag sequences classified each read in the raw sequencing data. A total of 21 RNA-seq data were used for human data, three samples each of LA, left atrial appendage (LAA), LV, pulmonary vein (PV), RA, RV, and SA. Fastp software (version 0.12.4, see reference [28]) was used for reading quality control and adapter removal [28]. In the raw data, the number of the generated pair-end reads per sample ranges between 49,720,696 to 368,429,512 reads (92,267,671 reads per sample on average). Further, we confirmed that Q30 > 90% is satisfied in all samples. Reads were aligned using STAR software (version 2.7.0a, see [29]). The fasta and gtf files of GRCh38/release105 obtained from the Ensemble database were used as reference genome and gene annotation information. The count values of each gene were quantified from the alignment results by featureCounts software (see [30]). We calculated the Transcripts Per Kilobase Million (TPM) values based on the calculated gene lengths and gene counts [31,32]. These values were converted by log2(intensity + 1) and used as gene expression intensity in subsequent analyses. As the quality check of this data, we compared our data with the public RNA-seq data in human RA, RV, LA, and LV samples (GSE112339 [33]). We confirmed the high correlation (Spearman’s correlation coefficient > 0.9) between the mean expression value of each region of our and previous data (Supplementary Figure S3).

### 2.6. The Evaluation of Similarities of Expression Intensity among Species or Regions

Hierarchical clustering was applied to the gene expression matrices of humans, mice, and rats to evaluate the similarity of regions. It was performed using the R package TCC’s clusterSample function with default setting [34]. The dendextend package was used to draw the results [35]. A comparison of transcriptome data among species was performed using gene ortholog relationships. Human–mouse, human–rat, and mouse–rat ortholog conversion tables were obtained from Ensemble Biomart. Using this table, scatter plots of gene expression profiles between species were created with one species as the *x*-axis and another species as the *y*-axis. We calculated Spearman’s correlation coefficient of expression values. For each gene of the *x*-axis species, an orthologous counterpart gene of the *y*-axis species was mapped. If more than one gene of the *y*-axis species corresponded to one gene of the *x*-axis species, the average value was applied.

### 2.7. Identification of Heart Region-Specific Expressed Genes

Heart region-specific genes were identified in each species. Three heart regions were targeted: LA, SA, and V. Here, LV and RV were grouped together as V. Genes whose expression values exceeded a certain threshold in at least one sample were used for subsequent analysis. These threshold values were defined as >1, >4, and >6 for humans, rats, and mice, respectively. They are determined based on the distribution of expression levels. For human RNA-seq data, one was added to all elements of the gene expression matrix before subsequent analysis to avoid zero values affecting the results. For each gene, differences in expression levels among regions were tested by analysis of variance (ANOVA), and *p*-values were calculated. The *p*-values were adjusted by the Benjamini–Hochberg (BH) method considering multiple testing problems. In addition to the statistical tests, the region specificity of LA, V, and SA was calculated as the specificity measure (SPM) value for each gene [36]. SPM is obtained by dividing the average expression value of a region by the absolute values of the average expression vector of three regions. SPM values range from zero to one; the larger the value, the more specific the region. A gene was defined as a specifically expressed gene at a region if SPM was above a certain threshold and BH adjusted *p*-value < 0.05. This threshold was defined as the 95th percentile point of the null SPM values calculated from one label permutation per gene. The same procedure as in the evaluation of similarities of expression intensity was used to calculate the correspondence of SPM values in human–mouse and human–rat. We conducted the clustering analysis for nine species-region pairs for the SPM values obtained for the human genes with orthologs in both rats and mice. The distance metric used in the clustering was the 1—Spearman’s correlation coefficient for SPM values, and the hclust function in R was used with default settings. The z-score normalized SPM was calculated by the standard normalization of SPM values for each species-region pair.

### 2.8. Comparison of Region-Specific Expressed Genes among Three Species

We conducted the biological interpretations of the region-specific gene sets (LA-specific, V-specific, and SA-specific) and compared them among species. First, we focused on commonly LA-specific, V-specific, or SA-specific gene sets in all three species. These are three gene sets: common LA-specific, common V-specific, and common SA-specific. This was defined as a region-specific gene in humans whose orthologous gene is also identically region-specific in both mice and rats. In addition, we focused on the species-selective region-specific gene set, which is a region-specific gene set for only one species. These are nine gene sets: human-selective LA-specific, human-selective V-specific, and human-selective SA-specific, mouse-selective LA-specific, mouse-selective V-specific, and mouse-selective SA-specific, rat-selective LA-specific, rat-selective V-specific, and rat-selective SA-specific. We focused on the transcription factor genes defined as the genes with Gene Ontology term GO:0003700 (DNA-binding transcription factor activity). Enrichment analysis for gene ontology was performed on the gene sets using the clusterProfiler package in R to identify the Gene Ontology (BH adjusted *p*-value < 0.05) associated with each gene set [37]. The background gene set of this enrichment analysis is all genes included in the region-specific gene analysis for the other two species. We used the R package (The R Project for Statistical Computing, https://www.r-project.org/) org.Hs.eg.db (version 3.13.0), org.Rn.eg.db (version 3.13.0), org.Mm.eg.db (version 3.13.0) as the databases for Gene Ontology and gene annotation.

### 2.9. Reverse Transcription-Polymerase Chain Reaction (RT-PCR)

Quantitative RT-PCR was performed using SYBR Green (BioRad, Hercules, CA, USA or Roche, Basel, Switzerland) on an ABI PRISM 799HT Sequence Detection System (Applied Biosystems, Waltham, MA, USA). PCR efficiency was evaluated by using a standard curve of four serial dilution points. Data were analyzed using Applied Biosystems software, and mRNA was normalized to the housekeeping genes, H1 or cardiac β-tubulin. All reactions were carried out in quadruplicate, and each average was in use. The PCR result measures the expression of the ventricle with arbitrary unit 1 and shows how many times more than the expression of the ventricle. Statistical significances of RT-PCR results were determined by an ordinary one-way analysis of variance (ANOVA) with Tukey’s multiple comparisons test, using GraphPad Prism ver. 9.0.0 (GraphPad Software Inc., San Diego, CA, USA).

## 3. Results

### 3.1. Classification of Cardiac Regions Based on Gene Expression Patterns

**Figure 1:**
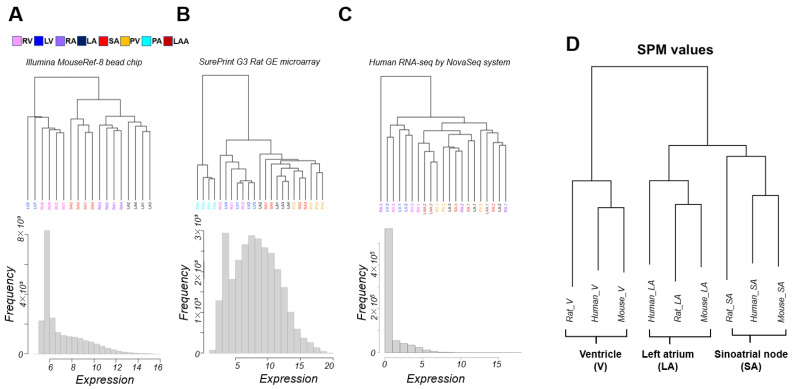
The clustering for the transcriptome samples and the distributions of expression intensity of gene expression intensities for mouse (**A**) and rat (**B**) array data (Illumina bead chip or SurePrint GE microarray) and (**C**) human RNA-seq data. (**D**) Interspecies comparisons of transcriptome data were performed using human gene ortholog relationships. For each gene, differences in expression levels between sites were tested by analysis of variance (ANOVA), and *p* values were calculated. For each gene, the site-specificity of the three sites was calculated as the specificity measure (SPM) value. Most of the analysis was performed on a single platform by SPM values. The results of hierarchical clustering of SPM values for site-specificity. Human genes with orthologs in mice and rats were targeted. The analysis was limited to the left atrium (LA), ventricles (V) (right ventricle (RV), and left ventricle (LV) data were combined), and sinoatrial nodes (SA), which are cardiac regions that are common in the transcriptome data. LAA; left atrial appendage, RA; right atrium, PA; pulmonary artery, PV; pulmonary vein. Each cardiopulmonary region is color-coded, with a corresponding panel in (**A**).

Microarray data of mice using Illumina’s Bead chip clustered gene expression patterns according to anatomic segments of the heart. The ventricles were further divided into the left ventricle (LV) and right ventricle (RV), and the atria into the right atrium (RA) and left atrium (LA) (Figure 1A). The rat microarray data using Agilent’s SurePrint included pulmonary artery (PA) and pulmonary vein (PV) samples and no RA sample compared to the mouse microarray sample. PA, being extracardiac tissue, is naturally clustered outside of the cardiac zone. Although clusters of ventricles were present, LV and RV were not cleanly separated. LA, SA, and PV were roughly clustered (Figure 1B).

On the other hand, unlike experimental animals, for which we controlled for strain and age in months, the human RNA-seq data had sizable individual variability, and it was difficult to achieve statistically superior clustering with only three samples for each cardiac region. The expression pattern was roughly divided into ventricular and non-ventricular, and ventricles were not separated into LV and RV (Figure 1C). We challenged academic discovery by cross-platform analysis of these unpublished transcriptome data, which were acquired for several different research projects. Samples were limited to LA, V, and SA; RA was not present in the rat microarray; LV and RV were computationally indistinguishable from the data in our possession. The specificity measure (SPM) values (see Section 2) were then calculated for each cardiac region (LA, V, SA) for a unified analysis of transcriptome data with different medians and units for expression levels. Cross-platform clustering of the SPM values of orthologous genes among mice, rats, and humans clustered the data by cardiac region rather than by platform (Figure 1D). Hierarchical clustering reflects the relative distance of relationships within the samples. In Figure 1A–C, the transcriptome sample in humans and rats did not separate well because the differences between regions were not very large compared to the differences between individuals. Our data processing method using SPM averages out the inter-individual differences, removes some of the platform differences and reflects the differences among regions in Figure 1D.

### 3.2. Interspecies Correlation of the Transcriptome

**Figure 2:**
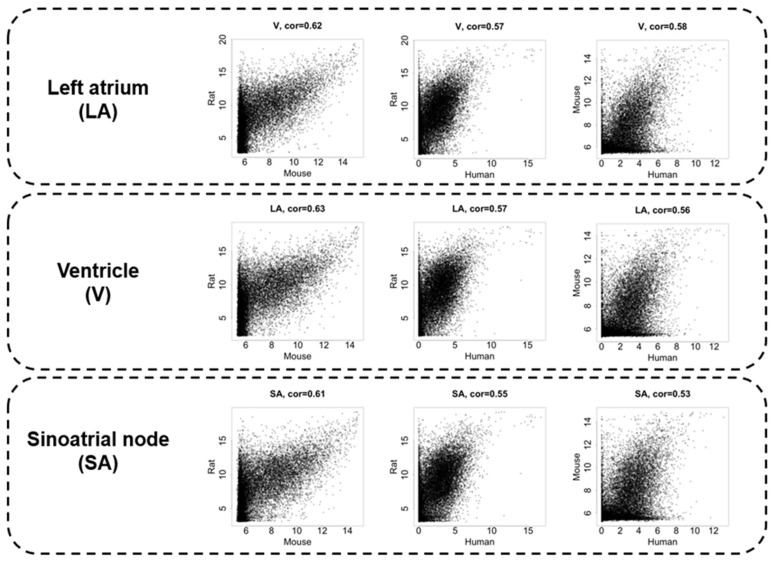
The expression intensity correlations between species are compared in the left atrium (LA), ventricle (V), and sinus node (SA), in order from top to bottom. The leftmost panels show the correlation between mice and rats, the middle panels show the correlation between rats and humans, and the rightmost panels show the correlation between humans and mice. Spearman’s correlation coefficient is displayed at the top of each graph.

Figure 2 displays scatter plots visualizing the relationship of gene expressions for each animal pair. The upper three panels show the LA, the middle three panels show the V, and the lower three panels show the SA from left to right the gene expression relationships in mouse–rat, human–mouse, and human–rat. The transcriptome data demonstrated a moderate correlation with Spearman’s correlation coefficient of 0.53–0.63 among the species. The correlation between mice and rats, both of which are rodents, was relatively high, being more than 0.6 in each cardiac region. Although rodents and humans are different order animals, the correlation coefficients between LA and V transcriptomes were 0.56–0.58. It is noteworthy that the SA transcriptome showed a correlation coefficient of less than 0.55 between rodents and humans and had the largest species difference in the heart region analyzed in this study (Figure 2 bottom, Supplementary Table S1).

### 3.3. Identification of Cardiac Region-Specific Transcription Factors

**Figure 3:**
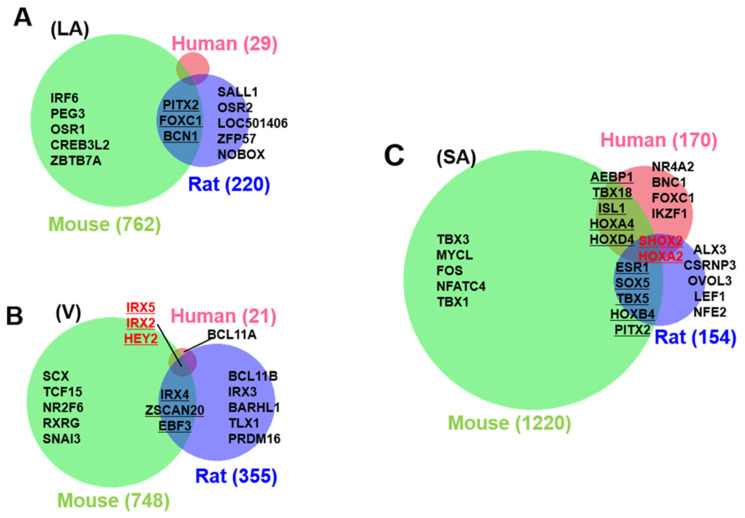
Cardiac region-specific genes. The number of left atrial (LA, (**A**)), ventricular (V, (**B**)), and sinoatrial node (SA, (**C**)) specific genes selected based on SPM values are indicated by numbers in parentheses beside the area-proportional Venn diagram. Animal species are color-coded as human (pink), mouse (green), and rat (blue). Transcription factors are shown as representatives of specific genes, with a maximum of five top-ranked transcription factors in each space. The rank is determined by the magnitude of the SPM value. Specific transcription factors that are conserved across more than two species are underlined; specific transcription factors that are conserved among the three species are shown in red.

**Figure 4:**
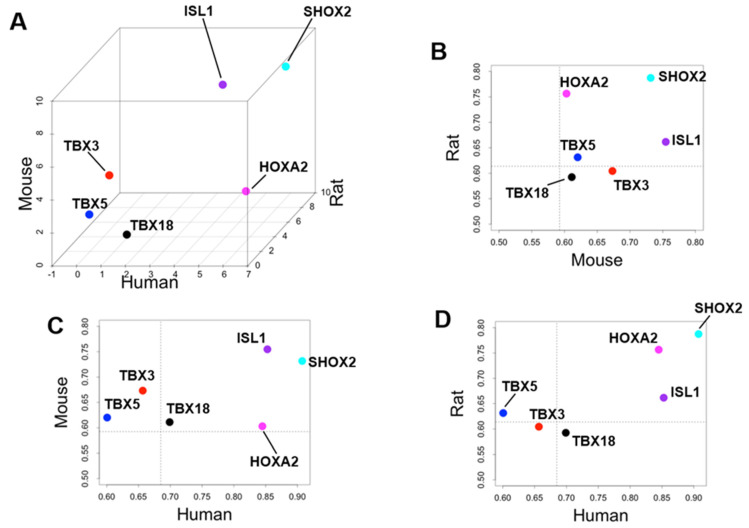
Specificity of six selected transcription factors with respect to sinoatrial nodes across three animal species. (**A**) Plot of z-score normalized specificity measure (SPM) values for three animal species: human on the *x*-axis, rat on the *y*-axis, and mouse on the *z*-axis. Scatter plots showing the relationship between (**B**) mouse–rat, (**C**) human–mouse, and (**D**) human–rat raw SPM values. The dotted lines indicate the threshold SPM values for specificity in this study. *SHOX2*, *HOXA2*, and *ISL1* exceed the threshold values in all species. *SHOX2*, *HOXA2*, and *ISL1* exceed the threshold values in all species.

**Figure 5:**
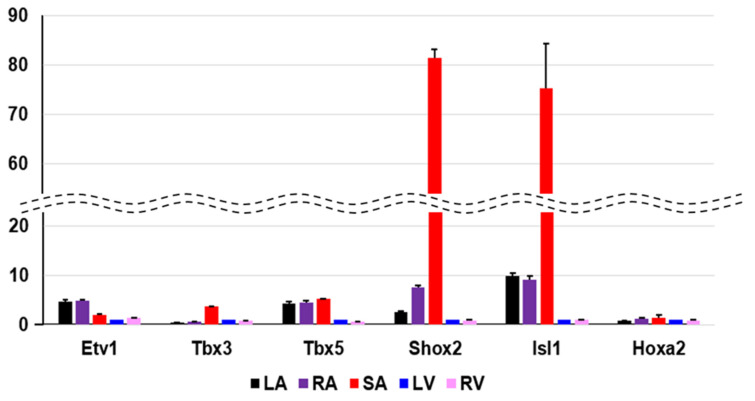
Quantitative PCR validation of selected transcripts. The PCR result measures the expression of the ventricle with arbitrary unit 1, and shows how many times more than the expression of the ventricle. In our transcriptome analysis, *Etv1* is atrium-specific, *Tbx3*, *Shox2* and *Isl1* are SA-specific genes in mice. With respect to *Hoxa2*, our specificity measure (SPM) criteria could not be confirmed. Each heart region is color-coded at the bottom. LA; left atrium, RA; right atrium, SA; sinoatrial node, LV; left ventricle, RV; right ventricle.

By calculating the SPM value, we can identify the cardiac region-specific genes in each platform (i.e., each animal species). 762 LA-specific genes were identified in mice, 220 in rats, and 29 in humans (Figure 3A). 748 V-specific genes were identified in mice, 355 in rats, and 21 in humans (Figure 3B). SA-specific genes were identified as 1220 in mice, 150 in rats, and 170 in humans (Figure 3C). The area-proportional Venn diagram in Figure 3 displays up to five transcription factors in each space, representing the specific genes of each animal species in each cardiac region. Some specific genes overlap between species; others do not. Three common LA-specific genes were identified across the species, *MYBPHL*, *IGFBPL1*, and *KCNJ3*; no transcription factors were among them (Figure 3A). There were 99 LA-specific genes common to mice and rats, and among these, nine transcription factors that dominantly regulate LA gene expression were found, including *PITX2*, *FOXC1*, *BCN1*, *SOX5*, *TBX5*, *FOXC2*, *ETV1*, *NR2F2*, and *ZBTB7C*. Figure 3A highlights three transcription factors (*PITX2*, *FOXC1*, and *BCN1*) that commonly rank in the top five SPM values in mouse and rat LA. 11 V-specific genes were identified throughout the species, of which *IRX5*, *IRX2*, and *HEY2* were transcription factors, and were considered to characterize the cardiac ventricle functionally or anatomically [38,39,40]. There were 81 V-specific genes common to mice and rats, of which three were transcription factors in *IRX4*, *ZSCAN20*, and *EBF3*. 17 SA-specific genes were common throughout the species, of which *SHOX2* and *HOXA2* were transcription factors. Transcription factors strongly expressed in SA have been studied for nearly 20 years as genes controlling cardiac automaticity. Not only *SHOX2* [41,42] and *HOXA2* [43] but also *TBX18* [44,45,46,47], *ISL1* [48,49], *TBX3* [46,50,51], and *TBX5* [52] have been reported. In our analysis, there were 81 SA-specific genes common to mice and humans, including seven transcription factors such as *SHOX2* and *HOXA2*, and *TBX18* and *ISL1* (Supplementary File S1). There were 80 SA-specific genes common to mice and rats, including six transcription factors (Supplementary File S1). There were 1075 mouse SA-specific genes not classified as human or rat SA-specific genes, 39 of which were transcription factors, and *TBX3* was among these 39 genes. *TBX5* was one of the atrium-specific genes common to mice and rats, as described above. Figure 4 plots the SPM values of these six transcription factors of interest in SA. Each axis of the 3D or 2D plot corresponds to the animal species from which the data originated, with the SPM values increasing from left to right or from bottom to top. Therefore, *SHOX2* in the upper right corner of the 3D plot in Figure 1A is a transcription factor with high SA specificity across species. Upon interpretation of the data, it is important to note that the selection of specific genes is based on an artificial threshold. Two continuous values, the *p*-value of ANOVA and the SPM value, are referenced to determine the threshold value. Not only *SHOX2* and *HOXA2*, which were selected as specific transcription factors by our criteria, but also *ISL1* satisfied the criteria for specific genes in terms of SPM value. The reason why *ISL1* cannot be concluded as an SA-specific transcription factor was due to its relatively low expression in rat SA, which failed to reach the threshold for the adjusted *p*-value (see Section 4). To confirm SA-specific transcription factors, quantitative PCR was performed using mouse samples. The relative PCR results in Figure 5 indicate how many times more than the ventricular expression level as an arbitrary unit. An analysis of variance accompanied by Tukey’s multiple comparisons test revealed that *Etv1* was predominantly expressed in LA and RA (*p* < 0.0001), while *Tbx3, Shox2*, and *Isl1* were predominantly expressed in SA (*p* < 0.0001). *Tbx5* was predominantly expressed in SA (*p* = 0.0002 vs. LA, *p* = 0.0032 vs. RA, *p* < 0.0001 vs. LV, RV). No significant differences in *Hoxa2* expression were observed.

### 3.4. Heart Region-Specific Genes across Species, and Ontology Analysis of Heart Region and Species-Specific Genes

**Figure 6:**
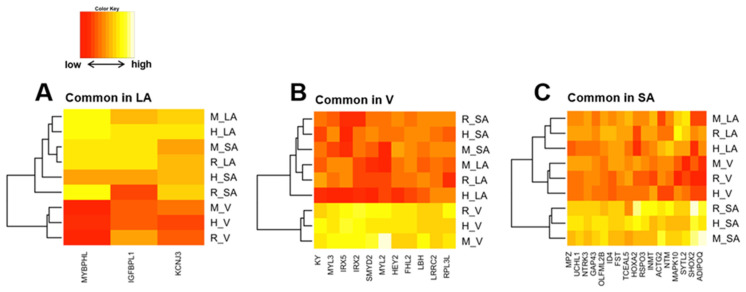
(**A**) is a heat map of z-score normalized SPM values in left atrial (LA)-specific genes that are common across species. Similarly, (**B**,**C**) show ventricle-specific gene groups and sinoatrial node (SA)-specific gene groups, respectively.

**Figure 7:**
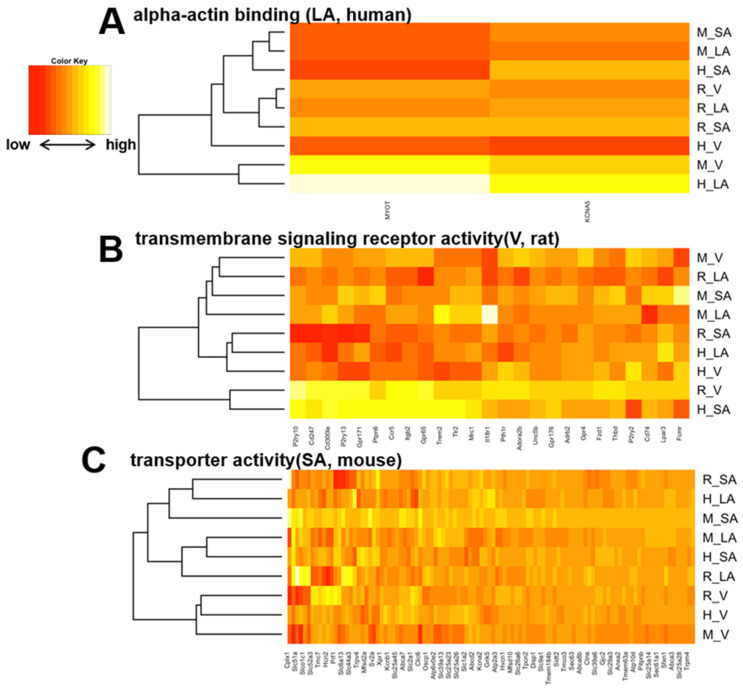
(**A**), A heat map of the human-selective LA-specific genes with the representative enriched gene ontology. Similarly, (**B**,**C**) show representative rat ventricle-specific ontologies and mouse SA-specific ontologies, respectively.

By calculating SPM values, we identified three atrial-specific, 11 ventricular-specific, and 17 SA-specific markers displayed in Figure 6A–C, respectively. These include *MYBPHL*, a known atrial marker ([53], Figure 6A), and *MYL3*, a known ventricular marker ([54], Figure 6B), which may have found novel cardiac region-specific markers. Unfortunately, *HCN4*, the most well-known marker of SA, was not on the mouse microarray chip and was therefore missing from our analysis; among the SA-specific genes, *GAP-43* is a marker of innervation in SA ([55,56]), suggesting similar innervation across species in SA. Ontology analysis revealed 70 ontologies that were cardiac region-specific and even species-specific (Supplementary File S2). Three examples of these are displayed in Figure 7A–C. Among human atrial-specific genes, *MYOT* and *KCNA5* both have the function of binding to the actin skeleton (Figure 7A), and although *KCNA5* is often considered an atrial marker, it is not an atrial-specific gene in mice or rats in our analysis. In rat ventricles, P2Y receptors (*P2ry10*, *P2yr13*, *P2yr2*), Toll-like receptors (*Tlr2*), G protein-coupled receptors (*Gpr171*, *Gpr65*, *Gpr176*), and IL18 receptors seem to be selectively expressed (Figure 7B). Although *HCN4*, an essential marker of SA, was dropped from the analysis as previously mentioned above, *HCN2* was a mouse-selective SA-specific gene (Figure 7C).

## 4. Discussions

### 4.1. Major Findings

This is the first study to analyze the transcriptomes of three separate cardiac regions in three different animal species. SPM values are a powerful normalization method, enabling region-specific gene analysis on cross-platform (Figure 1). The transcriptome, a comprehensive set of gene expression data, showed moderate correlation across species, but among the cardiac regions, SA showed the largest species differences (Figure 2, Supplementary Table S1). Therefore, we attempted to identify SA-specific transcription factors as a model for identifying special genes by cross-platform analysis using SPM values. Although transcription factors that characterize the SA function have been intensively investigated for a couple of decades, these studies technically had to take into account the differences among animal species. For examples, *TBX18* [44,45,46,47], *TBX3* [46,50,51], and *TBX5* [52,57,58] have been repeatedly reported as important transcription factors characterizing SA. *Tbx18* is identified by in situ hybridizations from the superior vena cava to 75% of the SA head, and *Tbx5* is expressed throughout the SA at 14.5 days of mouse embryonic development. Gene deletion of *Tbx18* results in abnormal SA morphology, while knockout of *Tbx3* results in no abnormal morphology but *Cx40* and *Cx43* gene, unrequired for SA function, is expressed in mice [46]. In 2013, Kapoor et al. selected *Tbx18* among five transcription factors, including *Shox2*, *Tbx3*, and *Tbx5*, as a transcription factor with a potent ability to form cardiac rhythm in screening using cultured cardiac cells [47]. They reported that the transfection of *Tbx18* can convert quiescent cells into pacemaker cells, and the *Tbx18* transfection strategy is becoming the basis of bioengineering to create bio-pacemakers [45]. *Tbx3* is believed to be important for the functional maturation of SA, as ectopic arrhythmias occur in conditionally knockout mice [51], and pacemaker currents are observed when *Tbx3* is overexpressed in stem cells [50].

*TBX5* is a transcription factor that regulates the electrical conduction system, rather than an SA-specific transcription factor. In human disease [57] and mouse models of *TBX5* deficiency [52,58], the PR interval on electrocardiogram is prolonged.

On the other hand, these three T-box transcription factors did not correspond to SA-specific genes in our criteria (Figure 3C and Supplementary File S1). *Tbx18* did not have a high enough SPM value in rat SAN, and *TBX3* did not have a high enough SPM value in both rat, and human SA. *TBX5* probably underlies the entire supraventricular area of the heart rather than the SA-specific (Figure 3C and Figure 5).

Interestingly, these T-box genes (*TBX18*, *TBX3*, and *TBX5*) exceed the criteria in mouse SA. In other words, our analysis reproduces the results of previous studies while at the same time suggesting that the previous studies have relied on mouse data. In our cross-species analysis, the only SA-specific transcription factors that are common across species are *SHOX2* and *HOXA2*, and the importance of *SHOX2* exceeds that of other transcription factors, especially given its high SPM value (Figure 3C and Figure 4, and Supplementary File S1). The SPM value is indicated as a measure of the degree of regional specificity. With this value, new candidate markers for atria, ventricles, and SA are shown in Figure 6. It is expected to be utilized in future studies. *KCNA5*, previously considered an atrial-specific ion channel [59], is an example. This gene is supposed to be a human-only atrial marker (Figure 6A). It is thought that the difficulty in developing atrial antiarrhythmic drugs targeting *KCNA5* is due to the fact that different K^+^ channels from *KCNA5* are the main source of outward current in experimental animals such as rats and mice [18].

### 4.2. Priority of SHOX2 over HOXA2 and ISL1

*SHOX2* is highly expressed in SA compared to other cardiac regions in every species, and both the ANOVA and SPM values strongly suggest its SA-specificity. *Shox2* was first reported as a transcription factor for SA differentiation 15 years ago. In situ hybridization identified *Shox2* as being expressed locally in the SA region, and *Shox2* knockout mice express *Nkx2.5*, a transcription factor required for differentiation of the working cardiac muscle, rather than SA, and promote expression of *Cx40* and *Cx43*, which interfere with SA function [41]. As a result, regular heartbeat is impaired in *Shox2* knockout mice in vivo [42]. In humans, mutations that disrupt *SHOX2* function also affect heart rhythm and are inherited as familial atrial fibrillation [60]. Our analysis reinforces that *Shox2* is a critically important gene for the healthy heartbeat. On the other hand, the calculation results concerning *HOXA2* and *ISL1* are not assured of reproducibility in the present analysis alone. *HOXA2* exhibits high SPM values in rat and human SA but only marginally above the threshold in mouse SA (Figure 4). In addition, significantly high expression in SA of this gene could not be confirmed by RT-PCR. It is not easy to define *HOXA2* as a common SA-specific transcription factor from our study. The number of samples and selected cardiac regions varies from experiment to experiment, and the threshold of the SPM value can change in future studies. *Hoxa2* has been previously mentioned once in other SA transcriptome analyses [43] and has rarely been studied as a gene involved in the cardiac beating. The function and locational information of *HOXA2* in the heart may be a research topic for future studies. *ISL1* had a high SPM value in SA in all species (Figure 4). In 2013, *Isl1* was identified as a binding target of *Shox2* [61]. Two years later, using a combination of genetically engineered mice and high-throughput analysis, two teams reported that *Isl1* is a genetic marker for SA and is involved in SA function [48,49]. *Isl1* may play a role in the maintenance of SA function comparable to *Shox2*. However, this well-known gene was not qualified as an SA-specific transcription factor in the current study because, as shown in Supplementary File S3, *ISL1* is not significantly higher in the rat expression intensity data. Furthermore, it is interesting that *HOXA2* and *ISL1* are most highly expressed in the pulmonary artery (PA), which is an extracardiac tissue (Supplementary File S3). Since the pulmonary artery is part of the secondary heart field in early development, *HOXA2* and *ISL1* may be genes whose expression was suppressed as the heart field differentiated [62,63]. In contrast, *SHOX2* was specifically and strongly expressed in the regions responsible for cardiac automaticity, including SA and pulmonary veins [64,65].

### 4.3. Validation of Our Analysis from Other Transcriptome Analysis

We checked the previous bulk-RNA-seq data set (GSE112339), which measured the transcriptome of human LA, LV, RV, and RA [33]. We compared the mean expression values of each region between our study and this previous study (Supplementary Figure S3). Spearman’s correlation coefficients are all greater than 0.9 (0.91 in LA, 0.90 in LV, 0.94 in RV, 0.93 in RA), indicating that although the sample size of our data is relatively small, it is sufficiently representative of previous data to be considered reliable. Recently, single-cell genomics analysis has become more prevalent in genomics analysis. Single-cell RNA-seq (scRNA-seq) data can provide information on cellular heterogeneity and quantify the cellular subset [66]. For example, the scRNA-seq study for the human heart reported V-common transcription factors *IRX5 and HEY2* as the transcription factors enriched in ventricular cardiomyocytes [67] as the same as our results. From a future perspective, it would be interesting to expand our computational workflow to single cell RNA-seq data and perform interspecies comparisons of heart single cell expression profiles. Such analysis may lead to further findings on the mechanisms behind the region-specific genes and heart biology interspecies differences.

### 4.4. Limitations

The present analysis is methodologically indirect since the raw data were normalized in multi-steps. The selection criteria for statistically significant specific genes were arbitrarily determined with consulting distributions of expression levels. For example, even though *TBX3* has recently been reported to be an important transcription factor for SA function in humans as well as mice [43], our analysis did not detect this gene as an SA-specific transcription factor. As such, our artificial threshold settings have limitations, and even if we fail to detect a gene as specific, the analytic results will not be universally reproduced. Despite this, the PCR data and the literature seem to support the certainty of our analysis to some extent, and the priority of *SHOX2* as SA-specific transcription factor may be established. Our strategy of using SPM values to identify unique genes is beneficial as long as we are aware of the limitations. From our view, the major limitation in the current study is that the number of the region-specific genes were small in humans. The cause could be the more considerable genetic or environmental diversity compared to laboratory animals, in addition to the limited sample size of human data, which leads to low detection power in human region-specific gene analysis. The region-specific genes with adjusted *p*-value < 0.05 were 43% in mice, and 36% in rats, compared to 6% in humans. In the case of human data analysis, the variation due to genetic or environmental effects is more significant than that of laboratory animals. Therefore, the detection power in the human data set would be smaller than laboratory animals, and the analysis of human samples requires a larger sample size than the analysis of laboratory animal samples to obtain the same detection power. Our analysis possibly did not contain enough region-specific human genes. In addition, because we limited our analysis to genes that are orthologous among the three species, we ignored genes expressed in mice and rats that are not orthologous. In other words, genes that are genuinely selective for mice and rats have been omitted. Furthermore, microarray platforms often do not carry determinant genes, and the absence of *HCN4* in the mouse array in our analysis damages the reliability of the SA data.

### 4.5. Future Perspectives

We identified species-dependent or cross-species information in the present study by comprehensively analyzing species differences in three cardiac regions. Such methods for refining information on genes of interest are not only species difference-based analyses but have also been used in previous analyses based on differences among animal strains. For example, Swindell et al. conducted the comparative transcriptomes between calorie-restricted and control mice using several strains of mice and reported the genes that respond in a strain-specific manner [68]. Another genomics study has reported the vital conservation of RNA editing sites among mouse strains, including wild-derived strains [69]. Such strain comparisons for the heart region-specific genes are a candidate application of the computational approach used in this study as a future perspective because it is already known that there are differences in heart size and function between strains of mice or rats. In a study comparing eight strains of mice, three strains had significantly reduced cardiac function after ex vivo ischemia, while two of these strains had significantly preserved cardiac function during in vivo acute hypoxia [70]. Twenty-three strains and a four-way crossbred strain of rats were examined for heart weight, and the strain differences in heart weight were significant, with the degree of genetic determination estimated to be 65% to 75% [71]. These differences between strains may be attributable to differences in genetic background. Considering that our analysis resulted in greater power to detect specific genes in laboratory animal samples than in human samples, it may be meaningful to identify common genes and strain-dependent genes based on strain differences.

## 5. Conclusions

In this study, we reanalyzed unpublished transcriptome data from three different platforms and employed the method of calculating the SPM values to examine species differences in transcriptomes from different cardiac regions. The analytic results were sufficiently robust to include findings that challenge existing knowledge. *TBX18*, *TBX3*, and *ISL1*, which were previously thought to be sinus node (SA)-specific transcription factors, are not classified as SA-specific transcription factors in humans or rats, and *SHOX2* and *HOX2A* are common SA marker candidates across species. In particular, the predominance of *SHOX2* as an SA-specific transcription factor was definitive. It was strongly likely that this transcription factor was essential for inducing biological pacemakers from stem cells. Thus, the identification of specific genes using SPM values can reveal tissue-specific and animal species-dependent genes. Novel cardiac region-specific genes may have been discovered in our study. It, thus, was suggested that what had been previously thought to be atrial and SA markers may be restricted to animal species such as humans and mice.

## Data Availability

The analytic results in this study are available on a request basis. All transcriptome data that we analyzed in the current study are uploaded into the GEO database. GSE200326, GSE203369, and GSE203367 are accession numbers for mouse Illumina array, rat Agilent SurePrint array, and human RNA-seq of heart transcriptome, respectively.

## Supplementary Materials

The following supporting information can be downloaded at: https://www.mdpi.com/article/10.3390/biom12060859/s1, Figure S1: Preparation of mouse sinoatrial node; Figure S2: Validation of SA node isolation technique; Figure S3: Validation of our human RNA-seq data by another study; Table S1: Correlation coefficient of transcriptomes among species; File S1: Sinoatrial node-specific transcription factors among species; File S2: Cardiac region-specific and species dependent ontologies. File S3: Rat microarray raw intensity data for six transcription factors. Reference [72] is cited in the Supplementary Materials.
