# Supplementary material for: Comparative Study of Transcriptome in the Hearts Isolated from Mice, Rats, and Humans
Source: Biomolecules. 2022 Jun 20;12(6):859. doi: 10.3390/biom12060859 (PMC9221511; doi:10.3390/biom12060859)
Supplement: Supplementary file 1 [file biomolecules-12-00859-s001.zip › Supplementary Figures.pdf]

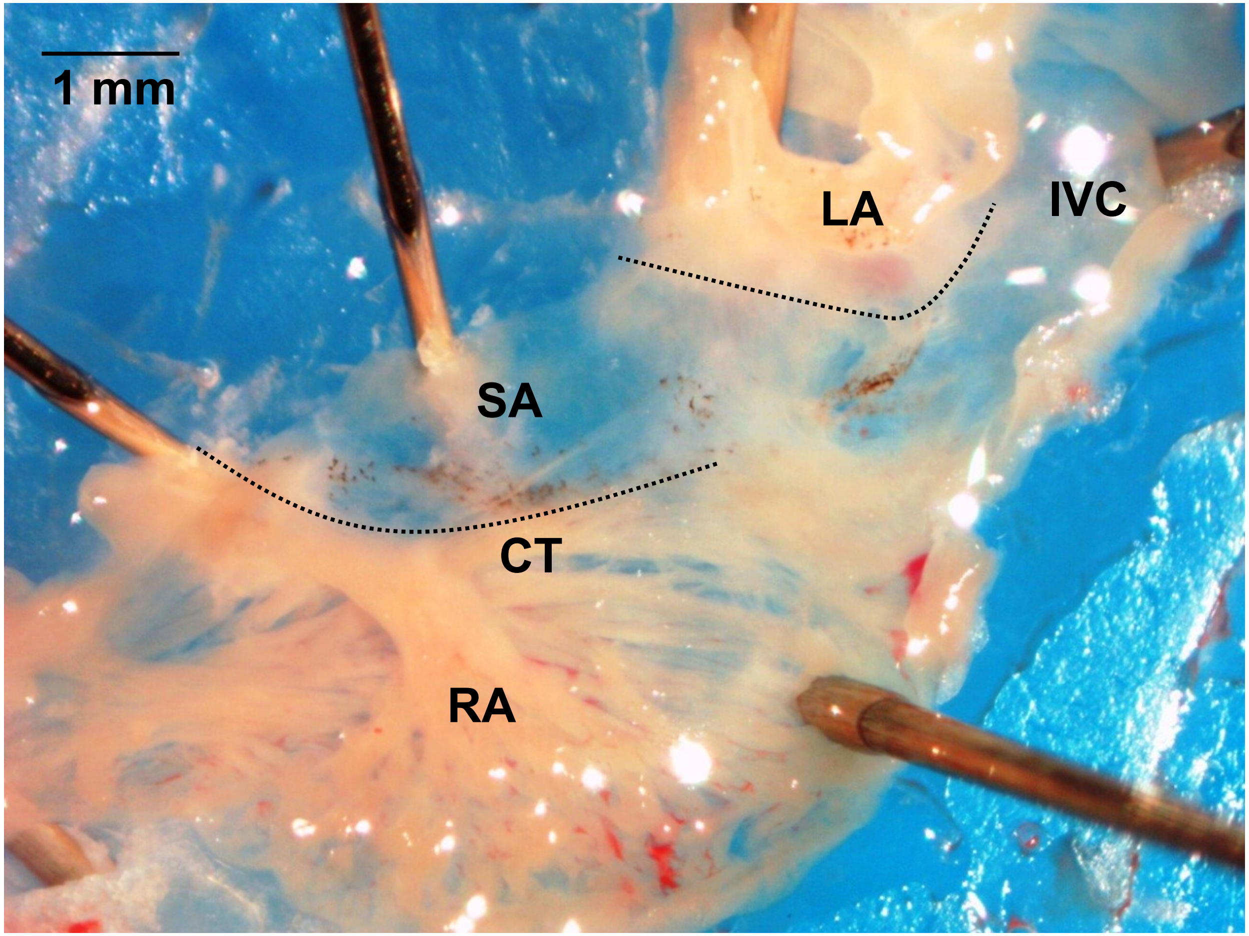

Supplementary Figure S1. The mouse sinoatrial node (SA) was acutely isolated according to classical landmarks (CT; crista terminalis, LA; left atrium, IVC; inferior vena cava, and RA; right atrium) [1].

[1] Aziz, Q.; Nobles, M.; Tinker, A. Acute Isolation of Cells from Murine Sino-atrial Node. *Bio Protoc.* **2020**, *10*, e3477. https://doi.org/10.21769/BioProtoc.3477.

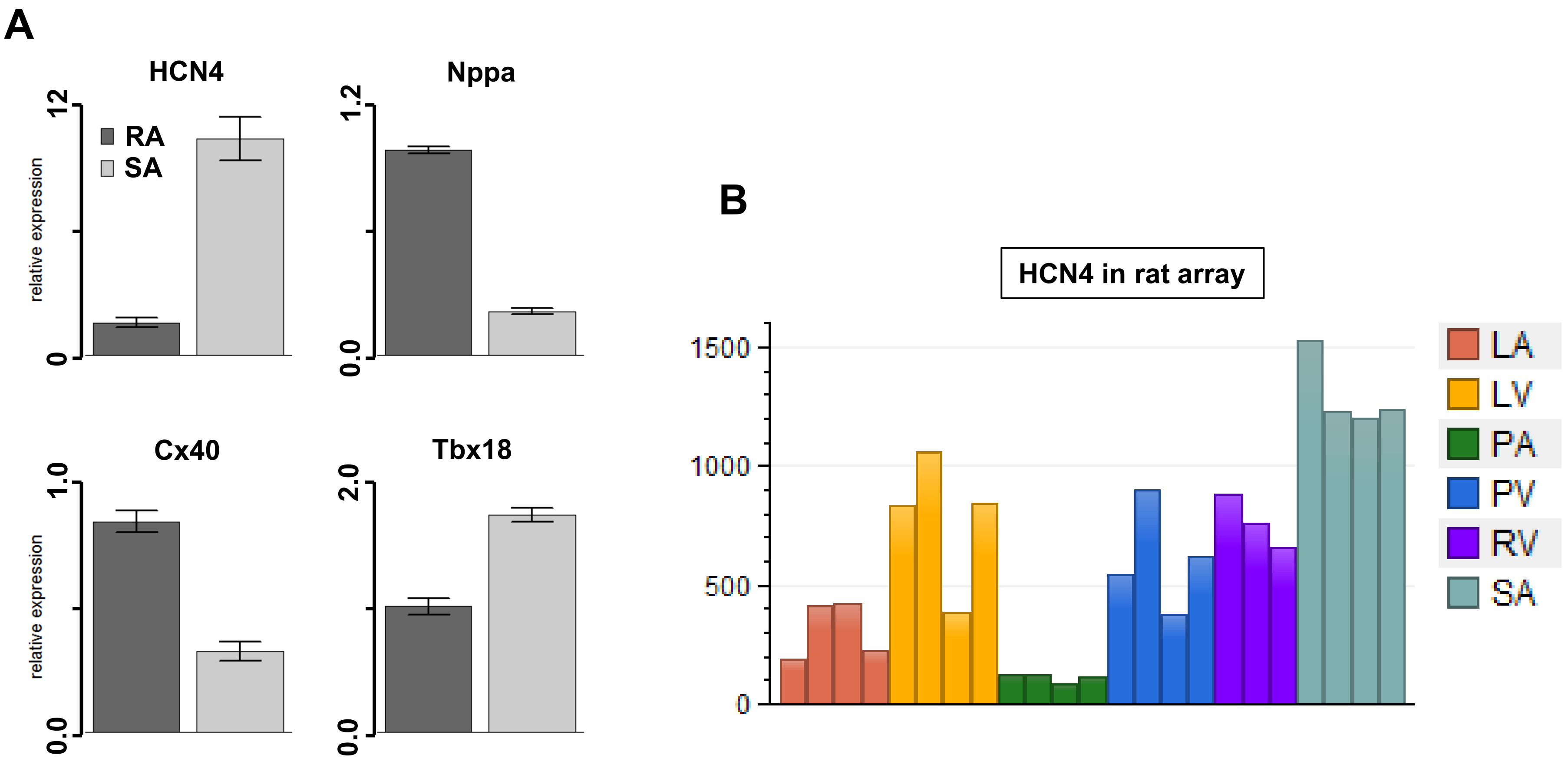

Supplementary Figure S2. Confirmation of sinus node (SA) isolation method (**A**), Quantitative PCR in mice confirms our SA isolation method. The right atrium (RA) and SA were sectioned at the border and the expression levels of HCN4, Nppa, Cx40, and Tbx18 in both tissues were compared; the RA expression levels were used as a unit; HCN4 and Tbx18 are markers of SA, while Nppa and Cx40 are markers of RA. (**B**), Expression of HCN4 in the Agilent rat array data. LA; left atrium, LV; left ventricle, PA; pulmonary artery, PV; pulmonary vein, RV; right ventricle.

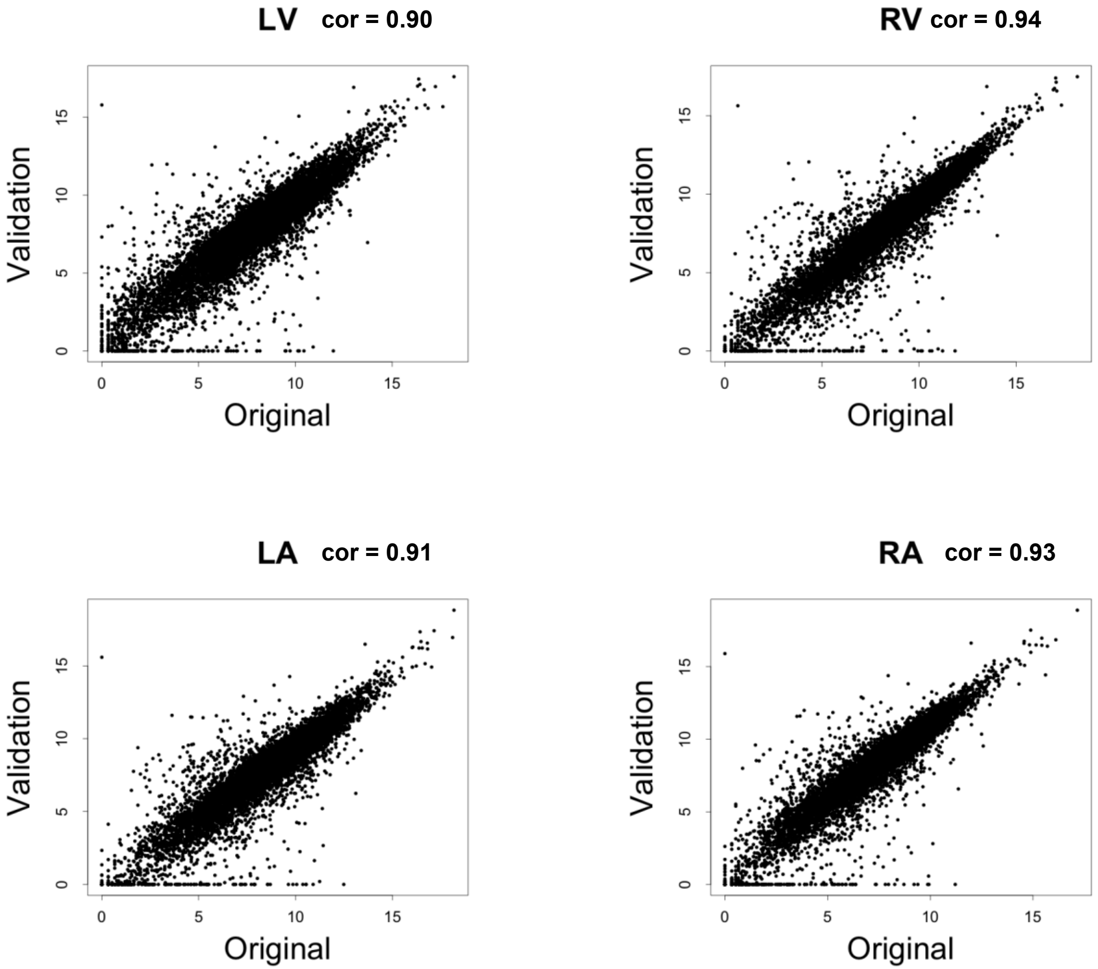

Supplementary Figure S3. Coplot of mean expression values for the four regions in the original and validation data. mean expression values were quantified from log2(count value + 1). Preprocessed count data were downloaded as validation data from GSE112339 and 10,000 genes were randomly plotted from the genes whose gene symbols matched in original data. The Spearman correlation coefficient for the each plot was 0.91 in LA, 0.90 in LV, 0.94 in RV, 0.93 in RA
