# Supplementary material for: Comparative Study of Transcriptome in the Hearts Isolated from Mice, Rats, and Humans
Source: Biomolecules. 2022 Jun 20;12(6):859. doi: 10.3390/biom12060859 (PMC9221511; doi:10.3390/biom12060859)
Supplement: Supplementary file 1 [file biomolecules-12-00859-s001.zip › Supplementary Table S1.pdf]

Supplementary Table S1. Correlation of transcriptomes among species by cardiac regions, ordered from highest to lowest Spearman's correlation coefficient.

Animal species	Paired species	Cardiac region	Correlation coefficient
Mouse	Rat	LA	0.627
Mouse	Rat	V	0.617
Mouse	Rat	SA	0.609
Human	Mouse	V	0.580
Human	Rat	V	0.573
Human	Rat	LA	0.571
Human	Mouse	LA	0.557
Human	Rat	SA	0.553
Human	Mouse	SA	0.532
